# The Neurophysiological Bases of the Impact of Neonicotinoid Pesticides on the Behaviour of Honeybees

**DOI:** 10.3390/insects10100344

**Published:** 2019-10-14

**Authors:** Amélie Cabirol, Albrecht Haase

**Affiliations:** 1 Center for Mind/Brain Sciences (CIMeC), University of Trento, piazza Manifattura 1, 38068 Rovereto, Italy; 2 Department of Physics, University of Trento, via Sommarive 14, 38123 Povo, Italy

**Keywords:** acetylcholine, *Apis mellifera*, neonicotinoids, neurobiology, plasticity

## Abstract

Acetylcholine is the main excitatory neurotransmitter in the honeybee brain and controls a wide range of behaviours that ensure the survival of the individuals and of the entire colony. Neonicotinoid pesticides target this neurotransmission pathway and can thereby affect the behaviours under its control, even at doses far below the toxicity limit. These sublethal effects of neonicotinoids on honeybee behaviours were suggested to be partly responsible for the decline in honeybee populations. However, the neural mechanisms by which neonicotinoids influence single behaviours are still unclear. This is mainly due to the heterogeneity of the exposure pathways, doses and durations between studies. Here, we provide a review of the state of the science in this field and highlight knowledge gaps that need to be closed. We describe the agonistic effects of neonicotinoids on neurons expressing the different nicotinic acetylcholine receptors and the resulting brain structural and functional changes, which are likely responsible for the behavioural alterations reported in bees exposed to neonicotinoids.

## 1. Introduction

In nearly 30 years since their first introduction, neonicotinoids have become the world’s most widely used insecticides [1]. Their low affinity for vertebrates seemed the major advantage in their toxicological profile [2]. However, harmful effect on pollinator insects started to be reported soon after their introduction, and the scientific evidence for this is now overwhelming [3]. Significant amounts of at least one neonicotinoid were found in 75% of honey samples collected worldwide [4]. Recently, the European Commission issued a ban on the outdoor use of three substances of this insecticide group (clothianidin, imidacloprid and thiamethoxam) [5,6,7]. Although the doses used in agriculture are sublethal, they are sufficient to alter the behaviour of individual bees and were shown to be partly responsible for the collapse of entire colonies [8,9,10].

Neonicotinoids are agonists of the insect nicotinic acetylcholine receptors (nAchRs), which are the main excitatory neurotransmitters [11,12]. When binding to nAchRs, neonicotinoids induce neuronal depolarisation and temporarily prevent the action of acetylcholine [11,13]. This results, e.g., in altered olfactory learning and memory abilities, which were shown to depend on cholinergic signalling [14,15]. In bees living in treated agricultural areas, neurons expressing nAchRs are stimulated by the neonicotinoids present in the nectar and pollen consumed [9]. This might trigger both activity-dependent synaptic plasticity and homeostatic plasticity, which maintains the stability of the neural network, as demonstrated in other species [16,17]. Yet, this question has received little attention in honeybee research.

While the impact of neonicotinoids on honeybee behaviour was described both in the field and under controlled laboratory conditions [14,18], the neural mechanisms involved have been scarcely investigated. The variety of doses, treatment durations, and exposure pathways used in scientific studies should enrich our understanding of neuronal plasticity, provided that data are numerous enough, but they still need to be put in context within a comparative analysis. Even the values of the field-realistic doses are controversial, as they vary from one geographical area to another, there are different uptake pathways, and the contaminated nectar or pollen is diluted within the total amount of nectar and pollen collected by the different bees [3,4,9].

After a brief description of the mode of action of neonicotinoids on nicotinic receptors, we consider the impact of different treatments with neonicotinoids on the brain of honeybees. The findings suggest that up to a certain level of concentration and exposure duration, the brain might be able to cope with neonicotinoids to maintain homeostasis. However, these levels still need to be identified [8,9,10]. By providing a summary of the literature on the neonicotinoid impact on honeybee brain, we aim at highlighting the current knowledge gaps, thereby orienting future research in this field.

## 2. Neonicotinoids as Agonists of nAchRs

Although all neonicotinoids are agonists of insect nAchRs, their affinity for these receptors and the neuronal response they elicit when binding to them depends on multiple factors such as the structures of the neonicotinoid itself and of nAchR, the brain region and the developmental stage of the individual [13,19,20].

### 2.1. Structure of nAchRs and Binding Properties

Nicotinic acetylcholine receptors are composed of five subunits, which assemble either in a homomeric or in a heteromeric combination [21]. These subunits are coded by 11 genes in the honeybee (subunits α1-9 and β1-2), and their diversity is further increased by alternative splicing [20]. There are multiple possible subunit combinations, which results in a great variety of nAchRs in the honeybee brain. The first studies on the mode of action of imidacloprid revealed its binding properties to nAchRs in cockroaches [22,23], which were later confirmed in the house fly [24]. The molecular structure of neonicotinoids and the subunits combination of nAchRs confer a high affinity and selectivity of neonicotinoids for insects nAchRs [13,25].

On the basis of the electrophysiological responses to the nAchR antagonist α-bungarotoxin (α-Bgt), nAchRs were categorized into α-Bgt-sensitive and α-Bgt-insensitive types [19]. This antagonist is often used in binding assays in competition with other molecular compounds, such as neonicotinoids, to assess their binding sites and affinity for nAchRs [19]. The binding properties of imidacloprid to nAchR subtypes differ between species (for a review, see [19]). In the *Drosophila* brain, imidacloprid was shown to bind to α-Bgt-insensitive nAchRs only [26], while in the aphid *Acyrthosiphon pisum*, imidacloprid could partially inhibit α-Bgt binding [27]. In cockroach, imidacloprid could bind both α-Bgt-sensitive and α-Bgt-insensitive nAchRs [28]. The subunit composition of nAchRs was shown to influence the binding of α-Bgt and imidacloprid in this insect [29,30]. Despite multiple evidence of honeybee sensitivity to neonicotinoids [9], our current understanding of the differential affinity of neonicotinoids for nAchR subtypes is still sparse in this insect.

### 2.2. Location of nAchRs in the Brain

**Figure 1:**
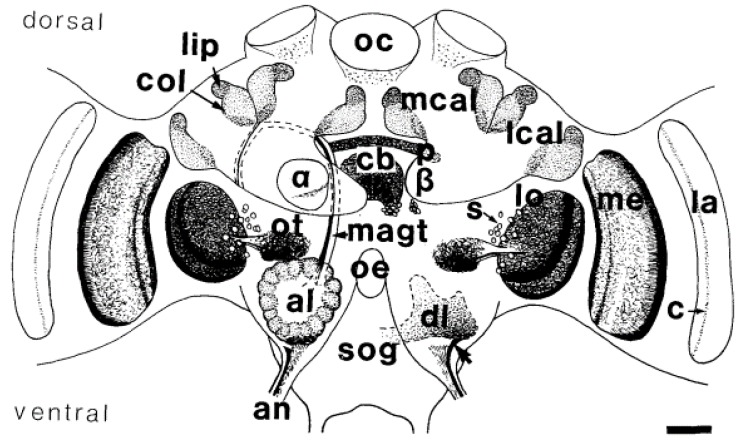
Schematic drawings of acetylcholinesterase (AchE) labelling in the neuropiles and some prominent groups of somata in the bee’s brain in a frontal view. The optic lobes lamina (la), medulla (me), and lobula (lo) display layered AChE staining. Weak AChE staining was found in the synaptic plexus of the ocelli (oc). The lobula is connected to the optic tubercle (ot). AChE-positive sensory fibres (arrow) of the antennal nerve project into the dorsal lobe (dl) and the suboesophageal ganglion (sog) below the oesophagus (oe). The median antennoglomerular tract (magt) connects the glomeruli of the antennal lobe (al) with the lip area (lip) of the median (mcal) and lateral calyx (Ical) of the mushroom bodies (MB). The collar (col) is a neuropilar compartment of the calyx receiving visual input. AChE-positive fibres leave the β-lobe (β) of the mushroom body. The central complex shows AChE activity in the pons (p), central body (cb) and a group of somata (s) ventrally to the central body. C-layer (c), α-lobe (α), antennal nerve (an). Scale = 100 µm (reproduced from [31] with permission by John Wiley and Sons).

Cholinergic synapses were detected in multiple regions of the honeybee brain using histochemical staining of acetylcholinesterase (AchE), the enzyme that hydrolyses acetylcholine after it has bound its post-synaptic receptor (Figure 1) [31]. AchE was found in primary sensory centres: the antennal lobes (ALs) for olfaction, the optic lobes for vision, and the gnathal ganglion for taste. A high proportion of neurons from these sensory centres, projecting into higher order brain centres such as the mushroom bodies (MBs) [32] and the central complex, was also positive for AchE activity [31]. The discrepancies in nAchR composition and binding properties complicate the localization of all nAchR subtypes in the honeybee brain [19]. The expression of nAchR subunits was shown to differ between brain regions [33]. The subunits α2, α8 and β1 were expressed both in the MB intrinsic neurons, called Kenyon cells (KCs), and in the ALs. The α7 subunit was found only in a subpopulation of KCs and in all AL neurons tested [33]. In another study, the expression of α2, α3 and α7 subunits was detected in AL neurons, but they were differentially expressed in KCs, depending on the KC subtype [34]. Interestingly, changes in the expression of nAchR subunits were reported at different developmental stages in both the European (*Apis mellifera*) and the Chinese (*Apis cerana*) honeybee, suggesting that neuronal responses to Ach and to neonicotinoids may vary with age [34,35]. The latter hypothesis still needs to be tested.

### 2.3. Neuronal Depolarization after Neonicotinoid Binding to nAchR

Electrophysiological experiments confirmed that information processing in the ALs and MBs was largely mediated by Ach [36,37]. The analysis of neuronal responses to Ach and imidacloprid in cultures of AL neurons confirmed the agonistic action of imidacloprid on at least two nAchR subtypes in this brain region [37,38].

Neonicotinoids show very different actions in cholinergic neurons. In most cases, imidacloprid acts as a partial agonist of nAchRs, e.g., inducing up to 43% of the maximum Ach-induced current (100 µM imidacloprid) in AL cell cultures [37], but it can act as a full agonist, e.g., in subpopulations of AL neurons (30 µM) [38]. Clothianidin showed even ‘super’ agonist action on nAChRs, evoking 56% higher currents than ACh (100 µM) in whole-cell patch-clamp recording from cholinergic neurons cultured from the central nervous system of third-instar *Drosophila* larvae [11]. Calcium imaging experiments performed on these cultures revealed that imidacloprid increased intracellular calcium levels dose-dependently [39].

The agonistic action of imidacloprid was confirmed in honeybee MB KCs by in vitro patch-clamp recordings [40,41]. In vitro calcium imaging revealed that imidacloprid and clothianidin activate different KC populations and distinct nAChRs [42]. Imidacloprid application to acutely isolated brains induced depolarization of KCs and had an antagonistic effect by blocking responses to Ach. Clothianidin application evoked a larger depolarization of KCs than imidacloprid [43]. A higher efficiency of clothianidin and acetamiprid in inducing neuronal depolarization compared to imidacloprid was also reported in cockroach neurons [44].

Acute oral exposure to imidacloprid (12 ng) was shown to increase neuronal activity in the MBs but not in the ALs of living bees [45]. The different expression of nAchR subunits between the MBs and the ALs might explain the variability of the neuronal response to nAchR agonists between these regions [33].

## 3. Neuronal Plasticity Following Neonicotinoid Exposure

### 3.1. Changes in Gene Expression Levels

Oral exposure of bees to various neonicotinoids was shown to increase the expression of genes coding for nAchR subunits. The neonicotinoid concentrations and the duration of exposure vary between studies, as well as the subunits tested for gene expression (Table 1). Oral exposure to imidacloprid (3 ppb), acetamiprid (80,000 ppb) and clothianidin (0.3 ppb) for 72 h increased the expression of α1 in the honeybee brain [46]. Thiamethoxam (1 ppb) increased α1 gene expression already after 48 h of treatment, but after 72 h, only the effect of 10 ppb was significant. Exposure to imidacloprid and thiamethoxam also induced the expression of α2 after 48 h, but here the effect vanished after 72 h of treatment [46]. Overexpression of the subunits α9 and β2 was reported after 10 days of exposure to thiamethoxam (10 ppb), but the expression of these subunits earlier in the treatment is unknown [47]. Changes in nAchR subunits expression after neonicotinoid exposure have also been reported in other insects [27,48,49]. They constitute a compensatory mechanism to the reduced Ach-sensitivity of the nAchRs present in the synapse. As long as bees are exposed to the pesticide, the expression levels of nAchR subunits in their brain seem modified [47]. 

Such homeostatic mechanisms of neuronal plasticity may be involved in the appearance of insects with resistance to neonicotinoids [54]. Another major adaptation is the increased expression of genes coding for detoxification enzymes after sublethal exposure to low doses of imidacloprid, thiacloprid and thiamethoxam in various insects including honeybees [47,48,49,55]. However, exposing bee larvae to imidacloprid downregulated the expression of these enzymes in the adults and might increase their sensitivity to xenobiotics [56].

### 3.2. Impact on Brain Function

**Figure 2:**
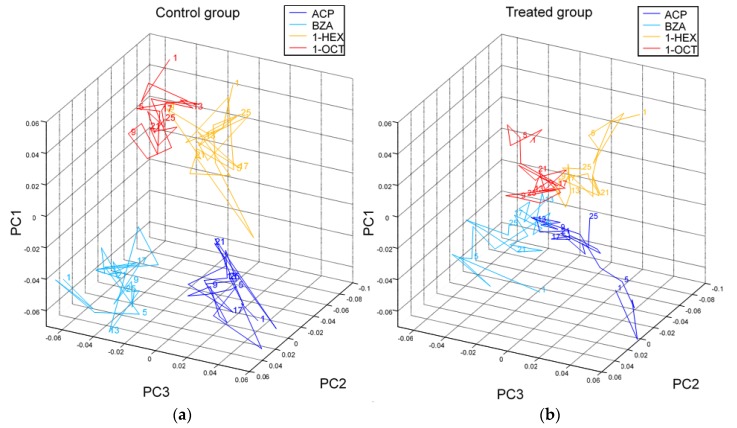
Odour representations in the AL: Glomerular response maps are displayed in principal components (PCs). The trajectories represent 25 odorant stimulus repetitions (marked by numbers along the trajectories) in control (**a**) and treated (**b**) bees. Imidacloprid was administered on average between trials 4.6 and 6.6 to the treated group (**b**), while, in the same time window, the control group (**a**) was administered Ringer’s solution; (*n* = 5 bees per group; ACP: acetophenone, shown in blue, BZA: benzaldehyde, in cyan, 1-HEX: 1-hexanol, in yellow, and 1-OCT: 1-octanol, in red). PCs and axes are identical for (**a**) and (**b**), allowing the comparison of odour distinguishability (reproduced from [61] CC BY 4.0).

The functional consequences of the increased expression in nAchR subunits suggest an elevated neuronal sensitivity to Ach. This hypothesis is supported by a study in bumblebees showing that oral exposure to sublethal doses of imidacloprid increased mitochondrial vulnerability to Ach after two days of treatment [57]. Yet, the immediate effects of neonicotinoids on the neuronal function are different. As mentioned above, they induce a neuronal depolarization and prevent Ach from binding to its receptors [37,43]. In the ALs, the synapse between olfactory receptor neurons coming from the antennae and the projection neurons projecting to higher order brain centres is cholinergic. Its activity can be recorded in vivo by injecting a fluorescent calcium-sensitive biomarker in the projection neuron tract and by observing changes in fluorescence via wide-field or two-photon microscopy [58,59]. Odorant-specific maps of activation were found in the ALs [60] and were shown to be affected by perfusing the brain with imidacloprid (10 µM) [61]. First, the mean response of projection neurons to different odorants decreased 1 min after the treatment, which is consistent with in vitro studies showing that responses to Ach were reduced after neonicotinoid exposure [40,43]. Second, the maps of activation representing the odorants in the AL were more similar after imidacloprid treatment, suggesting a decreased ability to discriminate odours. When plotting the odour-specific response maps in principle components, a proper collapse of these maps after imidacloprid administration is evident (Figure 2). Thus, neonicotinoid-induced impairments of olfactory learning and memory could actually be caused by this dysfunction of odour coding.

The consequences of chronic exposure to neonicotinoids on neuronal responses to sensory stimulations in various brain centres are obscure. Yet, the long-lasting increases in nAchR gene expression and mitochondrial vulnerability to Ach suggest an enhanced neuronal sensitivity to olfactory stimulations in bees chronically exposed to neonicotinoids [47,57].

In the visual system, imidacloprid reduced the firing of an interneuron involved in visual motion detection in the locust (*Locusta migratoria*), thereby altering its flight behaviour [62]. Consistently, imidacloprid reduced contrast and direction sensitivity of motion-sensitive neurons located in the lobula of the optic lobes of the hoverfly (*Eristalis tenax*) [63]. However, an almost complete loss of contrast as reported in the olfactory system (Figure 2) could not be observed, which could be dose-dependent or due to a different affinity of nAChRs to imidacloprid in the recorded visual pathway.

### 3.3. Impact on Brain Structure

**Figure 3:**
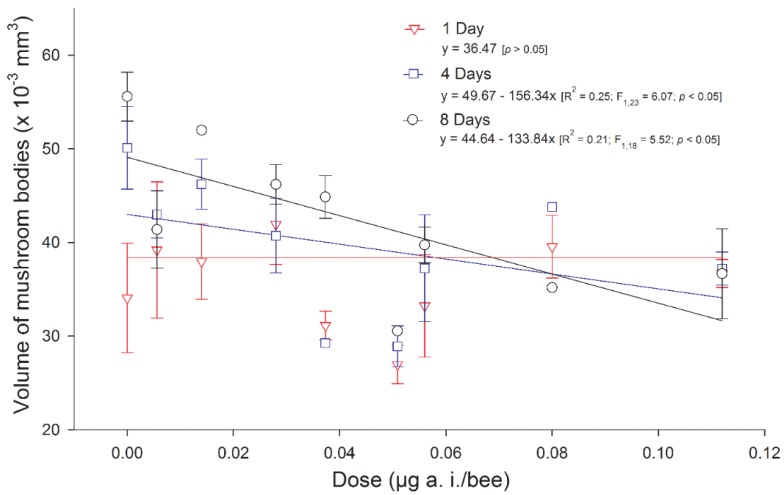
Mean volume of the mushroom bodies in the brains of 1-day-old (triangles), 4-day-old (squares) or 8-day-old (circles) stingless bee workers (*Melipona quadrifasciata anthidioides*) reared on imidacloprid-contaminated diets during the larval period. The vertical bars represent standard errors (reproduced from [66] CC BY).

As far as structural plasticity associated with neonicotinoid exposure is concerned, our knowledge is also extremely limited (Table 1). While the volume of the MBs was shown to increase during the first week of adulthood [64,65], it remained constant in native stingless bees (*Melipona quadrifasciata anthidioides*) chronically treated with imidacloprid in the larval stage [66] (Figure 3). In honeybees, chronic oral treatment with imidacloprid in the larval stage reduced the density of synaptic boutons in the mushroom bodies of 20-day-old adults [51]. Although the absolute values of the synaptic bouton density should be treated with caution because of a questionable counting method [67], these studies suggest that the brain is still affected for several days after the end of the treatment. Chronic oral exposure in the adult stage affected progressively the MB Kenyon cell structure, from mitochondrial alterations after 1 day of treatment to chromatin condensation after 4 days of treatment [53].

Studies on the impact of neonicotinoids on the honeybee brain were mainly focused on brain regions involved in olfactory learning and memory, namely, the antennal lobes and the mushroom bodies. This is because the involvement of nAchRs in the memorisation process of odorants associated with food reward was demonstrated in several studies [68,69,70]. Consistently, neonicotinoids at field-realistic concentrations were shown to affect olfactory learning and memory [14].

However, the mushroom bodies also receive input from visual afferents from the optic lobes [71], and behaviours relying on the visual sense, in particular navigation, are affected by neonicotinoid exposure [72,73,74]. The only available study on the structural plasticity of these regions shows that high doses of imidacloprid administered orally to Africanized honeybees increase cell death in the optic lobes and mushroom bodies, but little is known about the consequences of an exposure to field-realistic doses of neonicotinoids in the visual processing pathway in honeybees [52].

## 4. Conclusions

Taken together, scientific studies reveal the high complexity of the cholinergic system, which renders the prediction of neural responses to various levels of neonicotinoid exposure difficult. This is due to the diversity of nAchRs, their differential expression between brain regions and developmental stages and the variability in their affinity for and responses to agonists and antagonists, including neonicotinoids. Yet, our current knowledge already shows clearly that, from neuronal gene expression to volumetric changes in specific regions, neonicotinoids affect the brain. Further studies should now also investigate the neural changes persisting after the end of neonicotinoid exposure to reveal the full plastic potential of the brain and assess its ability to maintain homeostasis. Since the current agricultural use of neonicotinoids affects honeybee behaviour and the survival of entire colonies, it is likely that the doses and treatment durations used are beyond the limits of what single brain regions can cushion. Comparative studies with standardized doses and administration techniques should test this hypothesis. Finally, most research in this field is focused on the honeybee *A. mellifera*, while other bee species, whose cholinergic system might differ, are also endangered. Although this is justified by our deeper understanding of the honeybee neurophysiology, the study of the effects of neonicotinoids on the brains of other species is still necessary to understand how the cholinergic system has evolved and to preserve the biodiversity of pollinators.

## Figures and Tables

**Table 1:** Neuronal plasticity associated with chronic oral exposure to neonicotinoids in adult honeybees (* not European honeybees). Field-realistic doses based on [50] are highlighted in grey. nAchR: nicotinic acetylcholine receptor.

	Reference	Neonicotinoid	Treatment Duration	Minimal Dose	Effects
**Gene Expression**	[46]	Acetamiprid	24 h, 48 h, 72 h	80,000 ppb	Increased expression of nAchRα1 after 72 h
		Clothianidin		0.3 ppb	Increased expression of nAchRα1 after 72 h
		Imidacloprid		3 ppb	Increased expression of nAchRα1 after 72 h
				3 ppb	Increased expression of nAchRα2 after 48 h, but not after 72 h
		Thiametoxam		1 ppb	Increased expression of nAchRα1 after 48 h
				10 ppb	Increased expression of nAchRα1 after 72 h
				1 ppb	Increased expression of nAchRα2 after 48 h, but not after 72 h
	[47]	Thiametoxam	10 days	10 ppb	225 upregulated genes, 384 downregulated genes
					Increased expression of nAchRα9 and nAchRβ2
	[35] *	Acetamiprid	0.5 h, 1 h, 2 h, 3 h	10,000 ppb	Increased expression of nAchRβ1 and nAchRβ2 at 0.5 h
		Imidacloprid	1 h, 2 h, 3 h, 4 h	10,000 ppb	Decreased expression of nAchRβ1 and nAchRβ2 at 1–2 h
**Neuronal**	[51]	Imidacloprid	Larval stage (effects	10 ppb	Decreased synaptic bouton density in the MB lateral calyces
**Structure**			20 days after eclosion)	100 ppb	Decreased synaptic bouton density in the MB median calyces
	[52] *	Imidacloprid	1, 3, 5, 7 or 10 days	800 ppb	Increased cell death after 1 day in the optic lobes
				8100 ppb	Increased cell death after 1 day in the mushroom bodies
	[53]	Imidacloprid	1, 4, 8 days	14.6 ppb	Cellular alterations (mitochondria, chromatin, phagosomes)
